# Molecular Mechanisms of Apoptosis in HepaRG Cell Line Induced by Polyphyllin VI via the Fas Death Pathway and Mitochondrial-Dependent Pathway

**DOI:** 10.3390/toxins10050201

**Published:** 2018-05-15

**Authors:** Yi Liu, Xiaoxv Dong, Wenping Wang, Longtai You, Xingbin Yin, Chunjing Yang, Na Sai, Xin Leng, Jian Ni

**Affiliations:** 1 School of Chinese Materia Medica, Beijing University of Traditional Chinese Medicine, Beijing 100102, China; liuytcm@163.com (Y.L.); dxiaoxv@163.com (X.D.); wangwenp6@163.com (W.W.); ylt_svip@163.com (L.Y.); yxbtcm@163.com (X.Y.); ycj031927@163.com (C.Y.); yxsaina@126.com (N.S.); lengxtcm@163.com (X.L.); 2 Beijing Research Institute of Chinese Medicine, Beijing University of Traditional Chinese Medicine, Beijing 100102, China; 3 School of Pharmacy, Inner Mongolia Medical University, Hohhot 010110, China

**Keywords:** polyphyllin VI, hepatotoxicity, HepaRG cells, ROS, apoptosis

## Abstract

Polyphyllin VI, which is an active saponin, is mainly isolated from traditional medicinal plant *Paris polyphylla*, which causes liver damage in rats. In the present study, we aimed to explore the potential cytotoxicity of polyphyllin VI on the growth of HepaRG cells and to determine the molecular mechanism. The results revealed that polyphyllin VI changed cell morphology and induced apoptosis in HepaRG cells. Flow cytometric assay displayed that polyphyllin VI promoted the generation of reactive oxygen species (ROS), depolarized the mitochondrial membrane potential (MMP), and induced S phase cell cycle arrest by decreasing the expression of cyclin A2 and CDK2, while significantly increasing the expression of p21 protein. Polyphyllin VI induced the release of cytochrome c from the mitochondria to the cytosol and activated Fas, caspase-3, -8, -9, and PARP proteins. Pretreatment with NAC and Z-VAD-FMK (ROS scavenger and caspase inhibitor, respectively) on HepaRG cells increased the percentage of viable cells, which indicated that polyphyllin VI induced cell apoptosis through mitochondrial pathway by the generation of ROS and Fas death-dependent pathway. All of the effects are in dose- and time-dependent manners. Taken together, these findings emphasize the necessity of risk assessment to polyphyllin VI and offer an insight into polyphyllin VI-induced apoptosis of HepaRG cells.

## 1. Introduction

HepaRG cells, which were separated from a human hepatocellular carcinoma, exhibited several unique characteristics [1]. HepaRG cells can be differentiated into both the hepatocytes and the biliary epithelium by dimethyl sulfoxide. The cell expresses the similar metabolic characteristics when compared to human primary and the stronger growth capacity in vitro. According to these advantages, HepaRG plays a vital role in the assessment of drug-induced hepatotoxicity as a reliable surrogate to primary human hepatocytes for inquiring the drug-metabolism with activators of hepatic receptors (CAR, AhR, and PXR) [2,3]. Meanwhile, when compared with other liver cell lines, most of the major metabolizing cytochrome P450s were expressed high functional levels in HepaRG cells [4]. In addition, HepaRG can be cryopreserved without losing the specific biology function of normal hepatocytes [5]. These findings indicated that the HepaRG cell line could be a valid and optimal model for hepatotoxicity study in vitro.

**Figure 1:**
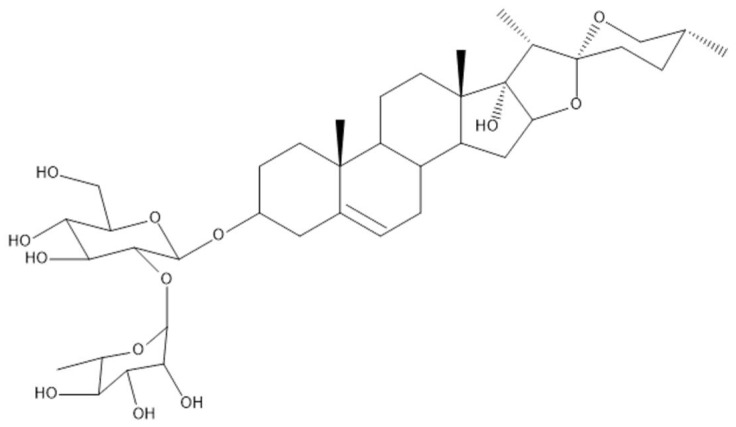
The Structure of polyphyllin VI.

Traditional Chinese Medicine (TCM) has a long application history as medicine and a dietary supplement [6]. *Paris polyphylla var. yunnanensis (Franchet) Handel-Mazzetti*, mainly distributed in East Asia, is a herbaceous perennial of family Melanthiaceae [7]. The rhizome of *Paris polyphylla* is usually used as an alexipharmic, detumescent, haemostatic, antifebrile, and demulcent agent [8]. Further phytochemistry and pharmacological studies confirmed that the Rhizoma Paridis Saponins (RPS), including polyphyllin I, II, VI, and VII are the main active ingredients, which determine the quality and the character of *Paris polyphylla*. In addition, free amino acid and phytosterol, phytoecdysone [9] are also included in *Paris polyphylla*. In consideration of its wide pharmacological activities, large quantities of pure compounds are worth studying further as chemical reference standards [10]. Recently, polyphyllin I, polyphyllin II, polyphyllin VII, and polyphyllin D have gained a lot of attention [11,12,13,14,15] while little research about polyphyllin VI has been investigated. As RPS was becoming the most popular antibacterial, analgesia, and anti-cancer agent, as far as growing safety evaluation and latent toxicity were concerned. Studies have shown that long-term use of *Paris polyphylla* could cause hepatic toxicity recently [16]. However, the pharmacological evaluation of the toxicity of *Paris polyphylla,* especially the toxicity of steroidal saponins, has been ignored. Previous studies showed that polyphyllin has significant cytotoxicity in several cells for example HepG2 cells [17,18], indicating that polyphyllin VI (structure as Figure 1) might be one of its main toxic components, but its toxic mechanism was unclear [19]. Therefore, this article aims to confirm the underlying cytotoxic effects of polyphyllin VI and to investigate the possible mechanisms and pathways on HepaRG cells.

**Figure 2:**
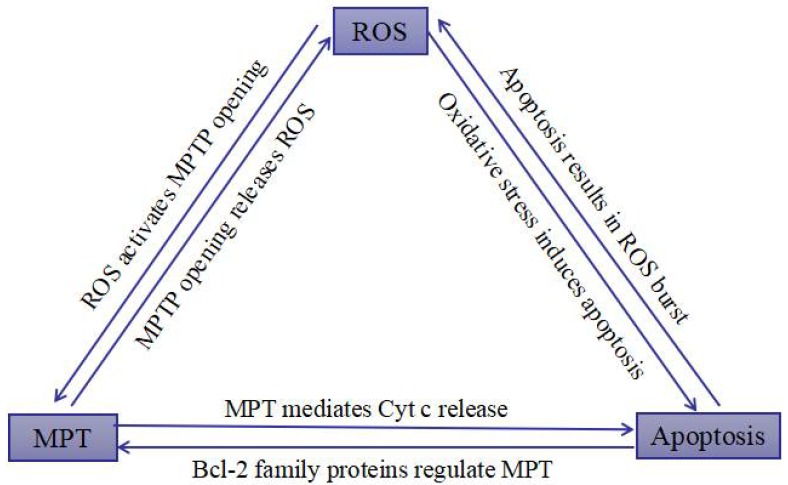
Reactive oxygen species (ROS), mitochondrial permeability transition (MPT), and mitochondrial apoptosis.

Apoptosis, which is characterized with nuclear fixation, cell shrinkage, DNA fragmentation, and cell membrane bubbles, is a tightly controlled cell death pattern [20,21]. The extrinsic pathway, named the death-receptor pathway, and the intrinsic pathway, named mitochondria-dependent pathway, are the two crucial pathways that are related to apoptosis [22]. The intrinsic pathway is mediated by Bcl-2 protease family which include apoptotic factors (Bax, Bim, Bak, Noxa, et al.) and anti-apoptotic factors (Bcl-2, Mel-1, Bcl-w, et al.). The survival or death of cells is determined by the ratio of Bax/Bcl-2 protein in response to an apoptotic stimulus [23]. High levels of ROS in mitochondria may depolarize the mitochondrial membrane, release several mitochondrial factors, and trigger caspase cascades [24]. There is a close relationship among ROS, mitochondrial permeability transition (MPT), and mitochondrial apoptosis, which forms an inseparable whole, as shown in Figure 2 [25]. Transient mitochondrial permeability transition pore (MPTP) opening is associated with ROS formation, and ROS is an effective activator of MPTP [26]. In the effective phase of apoptosis, the MPTP channel opens irreversibly and the permeability of the inner mitochondrial membrane increases, which cause the mitochondrial membrane potential (MMP) to decrease, oxidative phosphorylation on the respiratory chain uncoupled, ATP synthesis inhibited and ROS erupted largely. As a result, the cells are injured and are moved towards apoptosis [27,28,29]. Bcl-2 family proteins have important regulatory effect on MPTP. The over-expression of Bax can promote the opening of MPTP. In contrast, Bcl-2 can inhibit the continuous opening of the MPTP channel. Researches have shown that caspase can also induce MPTP to open and form a positive feedback amplification circuit of mitochondria-caspase-mitochondria in the apoptotic cells in order to amplify the apoptotic signal [30,31]. High concentration of ROS triggers oxidative stress in cells, ultimately causing the loss of mitochondrial function and cell apoptosis [32,33]. In addition, caspase can be activated by the death-receptor pathway, which covers the death receptors (Fas, TNFR1/2 and DR3/4/5) and associated ligands (FasL, TNF-α, TRAIL, and TWEAK). The objective of this article is to examine the toxicity of polyphyllin VI on HepaRG cells and to understand a potential ROS-associated mechanism.

Our group have focused on the hepatotoxicity of herbal medicine for several years. This research demonstrated the cytotoxic effect of polyphyllin VI on HepaRG cells and the underlying molecular mechanism. The results indicated that polyphyllin VI induced cell cycle arrest at S phase and apoptosis via both the Fas-death pathway and the mitochondrial pathway.

## 2. Results

### 2.1. Effects of Polyphyllin VI on Cytotoxicity

**Figure 3:**
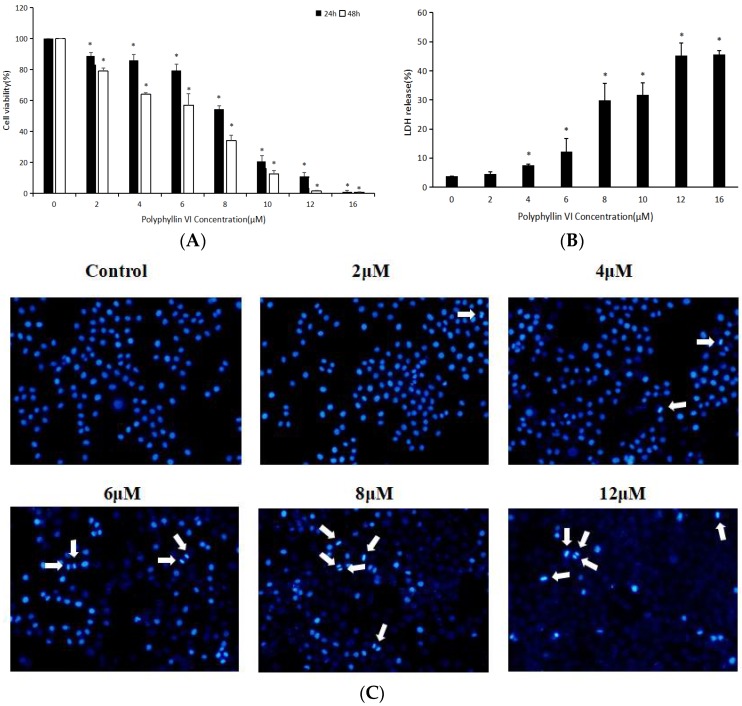
Effects of Polyphyllin VI on HepaRG cell viability and morphology. (**A**) 3-[4,5-dimethylthiazol-2-yl]-2,5 diphenyl tetrazolium bromide (MTT) assay. (**B**) Lactate dehydrogenase (LDH) assay. All the values are presented as mean ± S.D. of three independent experiments (* *p* < 0.05 vs. control). (**C**) Apoptotic cells were observed using a fluorescence microscope by DAPI staining.

The effects of polyphyllin VI on cell viability of HepaRG cells were determined by 3-[4,5-dimethylthiazol-2-yl]-2,5 diphenyl tetrazolium bromide (MTT) assay. HepaRG cells were incubated with polyphyllin VI (2, 4, 6, 8, 10, 12, 16 μM) for 24 and 48 h, respectively. As shown in Figure 3A, the treatment of HepaRG cells with polyphyllin VI resulted in a significant inhibition of cell viability in dose- and time-dependent manners. Different concentration treatments of polyphyllin VI induced reduction of HepaRG cell viability ranged from 88.90% to 1.07% after 24 h, and from 79.06% to 0.71% after 48 h. Lactate dehydrogenase (LDH), which is located in cytoplasm predominantly, was used to analyse cytotoxicity quantitatively. Consequently, the leakage of LDH indicates cell membrane injury. Results exhibited that the leakage of LDH occurred in HepaRG cells in a concentration-dependent manner following with different concentrations of polyphyllin VI for 24 h (Figure 3B). Moreover, to seek the potential molecular mechanisms about the polyphyllin VI on HepaRG cells, we chose polyphyllin VI at 2, 4, 6, 8, 12 μM in our further study.

### 2.2. Annexin V-FITC/PI Double-Staining Assay

**Figure 4:**
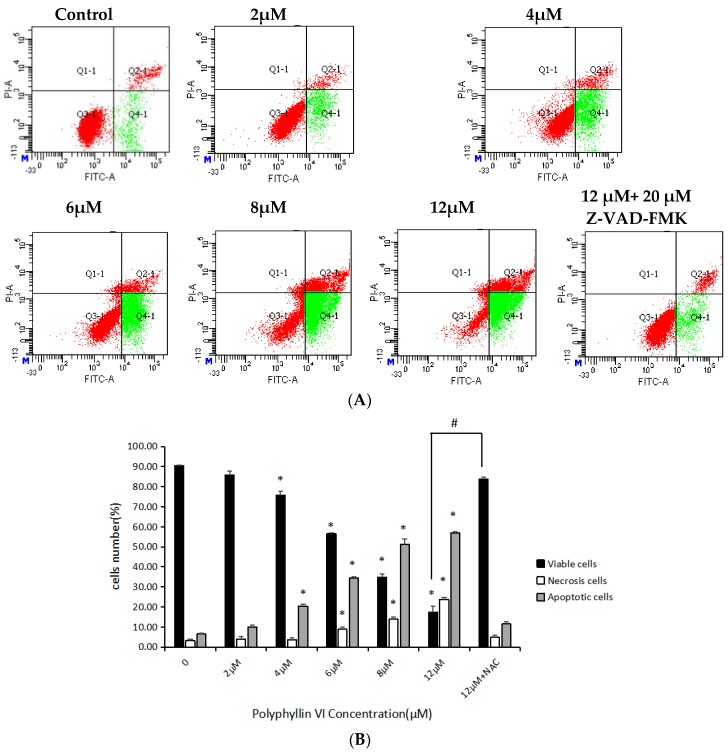
Polyphyllin VI induced apoptosis in HepaRG cells with or without Z-VAD-FMK pretreatment. (**A**) Flow cytometry detection of apoptosis. (**B**) The ratios of viable, early apoptosis and late apoptosis of cells after treatment with different concentrations of polyphyllin VI for 24 h. Data are expressed as means ± SEM (*n* = 3) * *p* < 0.05 vs. control; # *p* < 0.05 vs. polyphyllin VI 12 μM-treated group.

To enhance the results of MTT, Annexin V-FITC/PI double-staining was used to determine whether polyphyllin VI-induced cell death was caused by apoptosis. Annexin V-/PI+ cells symbolized the early apoptotic cells and Annexin V+/PI+ cells represented the late apoptosis cells. Results was shown in Figure 4, 24 h after 6 μM polyphyllin VI treatment, most HepaRG cells were undergoing apoptosis, whereas no significant apoptosis after being treated with 2 μM. The viable cells decreased in a concentration dependent manner. Furthermore, the ratio of early apoptotic cells increased evidently from 10.00% ± 1.04% to 56.93% ± 0.76%, along with a lower increase of late apoptotic and early necrotic cells from 3.97% ± 1.16% to 23.77% ± 1.04%. However, no obvious apoptosis was discovered in HepaRG cells that were pre-treated with Z-VAD-FMK, which is an caspase inhibitor. Z-VAD-FMK reversed the apoptosis of cells induced by polyphyllin VI significantly. The results indicated that polyphyllin VI induced HepaRG cells apoptosis possibly through the caspase-dependent pathway.

### 2.3. Effects of Polyphyllin VI on ROS

**Figure 5:**
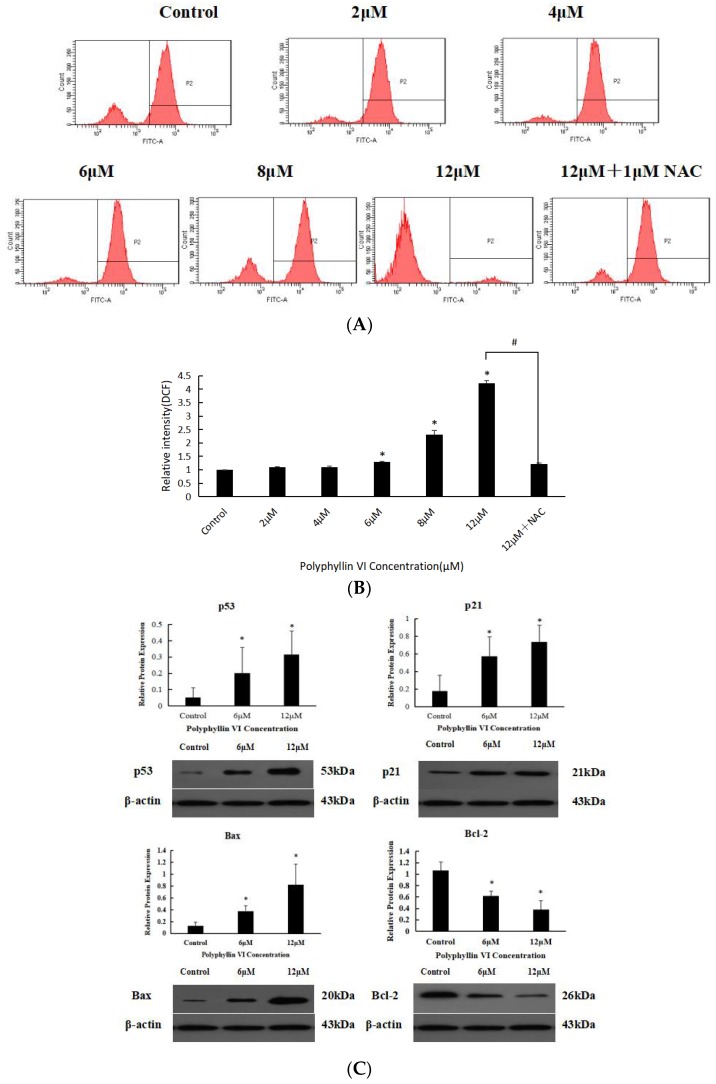
Effects of Polyphyllin VI on ROS generation in HepaRG cells. (**A**) The ROS production using flow cytometry with 2′7′-dichlorfluorescein diacetate (DCFH-DA). (**B**) The ratios of ROS after treatment with different concentrations of polyphyllin VI for 24 h, with or without pre-treatment with 1 mM NAC for 1 h. (**C**) The expression levels of p53, p21, Bax, and Bcl-2 determined by western blotting. β-Actin was used as internal controls. (* *p* < 0.05 vs. control).

The oxidative damage of DNA is caused by the interaction between DNA and reactive oxygen species (ROS), especially hydroxyl radicals [34]. It is confirmed that ROS production is an important factor in pro-apoptotic activities, and a high concentration of ROS triggers damage via the mitochondrial pathway. Since NAC is a ROS scavenger, we measured cellular ROS levels in NAC-existent and NAC-deficient HepaRG cells. As the results showed exactly in Figure 5, cellular ROS detected with DCF probes was elevated following with incremental concentrations of polyphyllin VI. In addition, the levels were significantly higher in NAC-deficient cells than in control cells (Figure 5B), and the existence of NAC inhibited the apoptosis of HepaRG cells. P53 protein strikes cell cycle arrest after cellular stress or DNA damage to provide time for repairing. To investigate the mechanism of polyphyllin VI-induced apoptosis in HepaRG cells, we analyzed the expression of p53, p21, Bcl-2, and Bax by western blotting. The results illustrated in Figure 5C, the expression levels of p53 and p21 were significantly increased. In particular, an increase in the Bax/Bcl-2 ratio revealed that the cellular resistance to apoptotic stimuli was decreased, resulting in apoptosis induction.

### 2.4. Role of MMP in Induction of Apoptosis by Polyphyllin VI

**Figure 6:**
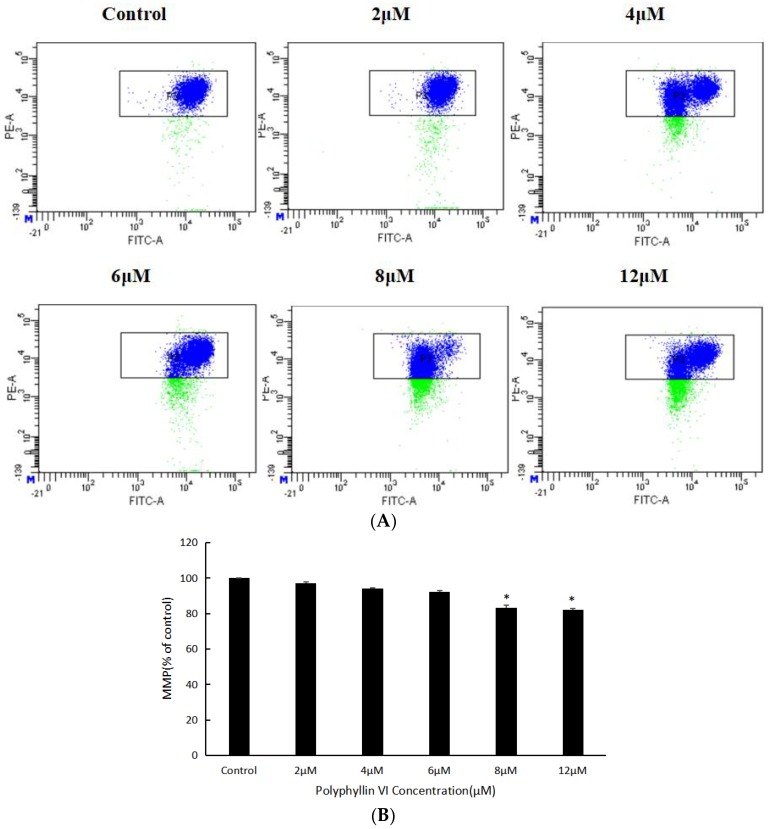

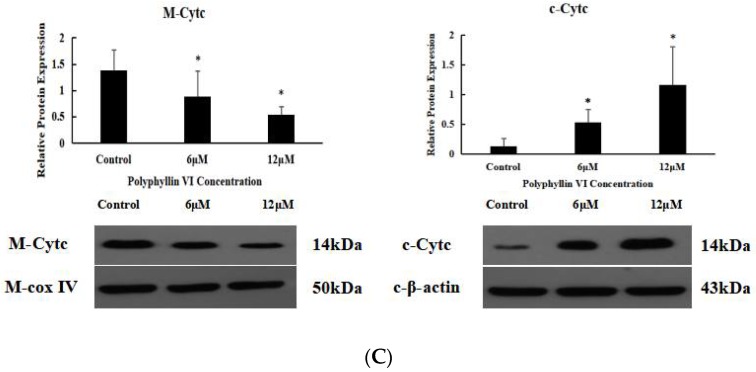
Effects of Polyphyllin VI on the levels of MMP in HepaRG cells. (**A**) The mitochondrial membrance potential (MMP) of HepaRG cells detected by flow cytometry with JC-1. (**B**) The loss of the MMP following incubation with polyphyllin VI for 24 h. (**C**) The expression levels of cyt c in mitochondrial and cytosol determined by western blotting. Actin was used as an internal control. β-Actin and COX IV were used as internal controls for the cytosolic and mitochondrial fractions, respectively. The data are expressed as means ± SEM for three independent experiments (* *p* < 0.05 vs. control).

To further illuminate the underlying mechanism of apoptosis on HepaRG, mitochondrial membrance potential (MMP) was measured. Reduction of the MMP is considered as an early marker of the apoptotic process. After cells were treated with polyphyllin VI for 24 h, the MMP fluorescent intensity obviously decreased in a dose-dependent manner when compared with control group (Figure 6A,B), indicating an imbalance of MMP and mitochondrial injury. The reduction of MMP implies the release of cytochrome c from the mitochondrion into the cytosol, which causes a caspase cascade activation for example Bcl-2 family proteins and then results in cell apoptosis. Besides, western blotting was conducted to detect the cytochrome c, both in mitochondrial and cytochrome fractions. Results revealed that the expression level of cytochrome c was significantly decreased in the mitochondria and correspondingly increased in the cytosol after polyphyllin VI treatment (Figure 6C). These experimental data explained that the mitochondrial dysfunction in HepaRG cells is potentially involved in the polyphyllin VI-induced apoptosis.

### 2.5. Polyphyllin VI Induced Cell Cycle Arrest

**Figure 7:**
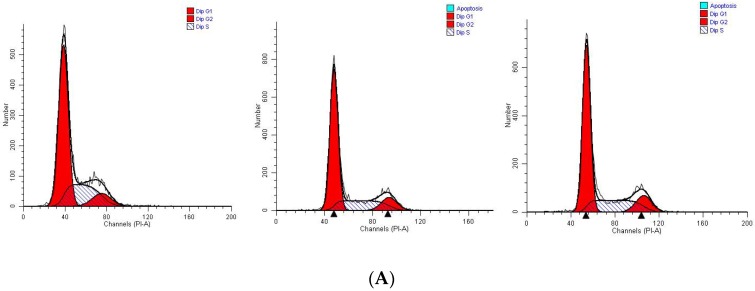

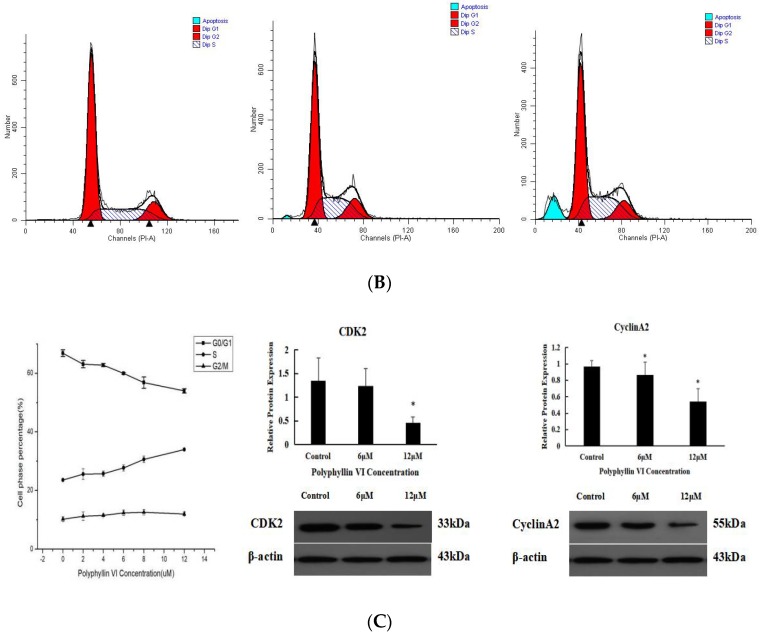
The effect of polyphyllin VI on the cell cycle and the expression of related regulators in HepaRG cells. (**A**) The stained cells were measured by flow cytometry. (**B**) The proportion of cells in each phase of the cell-cycle treated with polyphyllin VI for 24 h. (**C**) The expression levels of p53, p21, cyclin A2, and CDK2 were determined by western blotting. Actin was used as an internal control. The data was represented as the mean ± SEM for three independent experiments (*p < 0.05 vs. control).

To illuminate the polyphyllin VI-induced suppression of cell growth is relevant to cell apoptosis, we investigate the cell cycle of polyphyllin VI-treated HepaRG cells using flow cytometry. The results shown a mild increase (*p* < 0.05) in S phase and an prominent decrease(*p* < 0.05) in G0/G1 phase as the concentration increases (Figure 7A,B). After 24 h of polyphyllin VI exposure, the ratios of cells in the S and G0/G1 phase changed from 23.62% ± 0.14% to 34.01% ± 0.32%, 66.88% ± 1.15% to 54.00% ± 0.71%, respectively, when compared with the control group. In other words, the data demonstrated that polyphyllin VI could arrest HepaRG cells at S phase. What is more, our group investigated the protein levels that were related to the S-phase progression to obtain the further insight into the effect of polyphyllin VI on the cell-cycle distribution. As illustrated in Figure 7C, polyphyllin VI treatment down-regulated the expression levels of CDK2, cyclin A2 in HepaRG cells. Furthermore, p53, which is a tumor suppressor protein, regulates the cell cycle distribution and its target gene, p21, directly inhibits the expression of cyclin A2 and CDK2. These results reveal that polyphyllin VI blocked the cell cycle progression through altering the expression of cell cycle regulators.

### 2.6. The Expression Levels of Apoptosis-Related Protein

**Figure 8:**
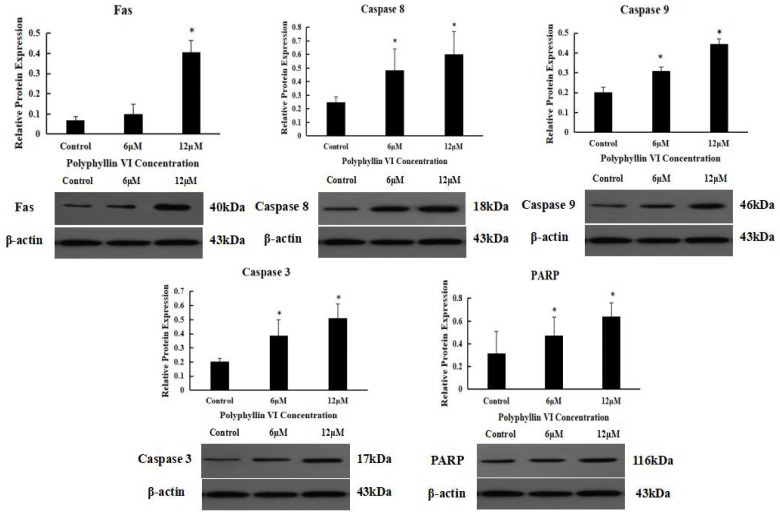
Effects of polyphyllin VI on the expression of apoptosis-related proteins.

For further investigating the possible mechanism of polyphyllin VI-mediated apoptosis, HepaRG cells were incubated with or without polyphyllin VI before the cells were harvested. Then, activities of apoptosis-related protein (Fas, caspase-8, -9 and -3, PARP) were analyzed using western blot analysis. As shown in Figure 8, the expression of Fas, which is a well-known death-receptor, was increased after being incubated with 6 μM and 12 μM polyphyllin VI for 24 h when compared with control cells. Polyphyllin VI treatment increased the expression of PARP, as a substrate of caspase-3, which results in the biochemical and morphological changes in cells. The expression of caspase-3, -8, and -9 were increased in the response to polyphyllin VI, which indicated that polyphyllin VI activated the caspases and elicited death signals. All of these results declared that polyphyllin VI triggered apoptosis in HepaRG cells through both extrinsic and intrinsic apoptotic pathways.

## 3. Discussion

With the development of multi-level and multi-target research of Traditional Chinese Medicine (TCM) in recent years, the toxicity of Chinese herbs has attracted more and more attention. Grasping the toxicity of TCM comprehensively and understanding the hepatotoxicity of TCM correctly were of great significance to evaluate the safety of New Drugs’ R&D and to reduce the risk of drug-induced liver damage. Several reports about the clinic toxicity of *Paris polyphylla* have been published at home and abroad. The sub-acute toxicity experiments confirmed that, when polyphyllin was used at 2.65 g/kg, rats would have obvious adverse reactions, or even death [35]. In addition, it was reported that a single-dose of polyphyllin could cause adverse reactions and even death of mice, and the oral acute toxicity LD_50_ was 2182.4 mg/kg [36]. However, the toxic monomer component and its hepatotoxicity mechanism were unclear until now. Therefore, this paper aims to study the hepatotoxicity and its underlying mechanism of the main component-polyphyllin VI. In our study, MTT assay and LDH assays showed that polyphyllin VI effectively induced the apoptosis of HepaRG cells in a time- and dose-dependent manner. DAPI-staining studies further proved that polyphyllin VI impaired cell membrane integrity and increased cell apoptosis of HepaRG cells. Besides, our experiments confirmed that the ROS levels were dose-dependently increased, indicating the existence of oxidative stress. Furthermore, our data suggested that polyphyllin VI induced the disorder of MMP with an persistent release of cytochrome c from the mitochondria into the cytosol. In western blot analysis, polyphyllin VI significantly increased p53 expression and the ratio of Bax/Bcl-2 in HepaRG cells, suggesting that polyphyllin VI induced mitochondrial membrane permeabilization, which discharged cytochrome c to the cytosol and activated of caspase-9 [37,38]. These results led to the activation of the executioner caspase-3 and PARP eventually. In addition, the expression of Fas was up-regulated, which activated initiator caspase-8 and caspase-3. These results gave rise to apoptosis [39].

**Figure 9:**
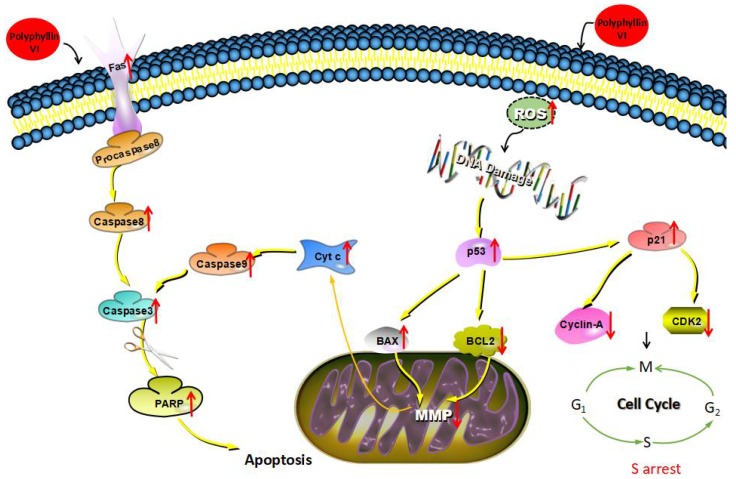
The extrinsic and intrinsic signal pathway of apoptosis induced by polyphyllin VI.

Apoptosis can be induced either via an extrinsic manner or an intrinsic manner. The former is triggered by the binding of apoptosis-inducing ligands to cell surface receptors, whereas the latter is determined by pro-apoptotic and anti-apoptotic proteins in the mitochondria [40]. The oxidative damage of DNA is caused by the interaction between DNA and reactive oxygen species (ROS), especially hydroxyl radicals. P53 protein caused cell cycle arrest following with the DNA damage in order to provide time for self-mediated apoptosis or repairing of damage. The Bcl-2 family conduced to apoptosis regulation, including Bcl-2 and Bax. In particular, antiapoptotic members, such as Bcl-2, act to prevent or delay cell death, whereas the pro-apoptotic Bax promote apoptosis. The ratio of Bax/Bcl-2 protein determines the survival or death of cells in the presence of apoptotic stimulus. In addition, p53, which is a tumor suppressor protein, regulates the cell cycle, and its target gene, p21, inhibits the expression of cyclin A2 and CDK2 directly. The signal of the CDK/cyclin complex is received by a series of cascade reactions after the DNA damage, which determines the course of cell cycle. CDK2 is the key kinase that initiates DNA replication. The Thr160 site phosphoric acids is the active form of CDK2. CDK2 is also a necessary condition for the running of S phase. Cyclin A2 appears in the late G1 and can be combined with CDK2. The western blot results also showed that polyphyllin VI could significantly inhibit the activities of CDK2 and cyclin A2 kinase when compared with control groups. HepaRG cells that were treated with polyphyllin VI activated the S-phase checkpoints, causing cell cycle-phase arrest. Since polyphyllin VI has a significant blocking effect on the S-phase of cells, it is considered that polyphyllin VI may cause cell cycle to be blocked by causing DNA damage. In the present study, polyphyllin VI treatment caused an apparent dose-dependent increase in the expression of Fas, which was a death receptor. The expression levels of cleaved caspases-3, -8 and PARP were increased, respectively, as demonstrated through western blotting. The intrinsic or mitochondrial pathway is commenced with the release of mitochondrial cytochrome c, which led to the activation of caspase-9 and caspase-3 [41]. The ratio of anti-apoptotic Bcl-2 protein and pro-apoptotic Bax plays a significant role in the release of mitochondrial cytochrome c [37]. Given that polyphyllin VI inhibited the growth of HepaRG cells, further investigation was performed to establish whether the increased apoptosis was linked to the increased expression of apoptotic genes. Our results revealed that treating HepaRG cells with polyphyllin VI decreased the ratio of Bcl-2/Bax, while it increased the expression of cytochrome c in the cytosol, cleaved caspase-9, and caspase-3. These results illustrated that the death-receptor pathway and mitochondrial pathway are the underlying mechanism of polyphyllin VI-induced apoptosis (as shown in Figure 9). Briefly, polyphyllin VI-induced cell apoptosis may have gone through the extrinsic death receptor signaling pathway involving the Fas and Fas-L, then activating caspase-8, before then either direct to activate caspase-3 for causing apoptosis or through ROS production, dysfunction of mitochondria, cytochrome c release from mitochondrial, and then the activation of caspase-3 through the intrinsic mitochondria-mediated pathway in HepaRG cells. The two pathways that can trigger caspase cascades ultimately in different forms play important roles in apoptosis induction under physiologic and pathologic conditions. Thus, polyphyllin VI can be considered to be one of the major hepatotoxic substances in the herbal medicine that was derived from *Paris polyphylla*.

In conclusion, these results suggested that polyphyllin VI exerted cytotoxic effects on HepaRG cell lines mainly through apoptosis that is mediated by the Fas–Fas ligand and the mitochondrial pathway. Previous studies of polyphyllin VI focused on its pharmacologic actions, especially the effect on lung cancer cells [42], while its side or toxic effects were often neglected. Notably, the present study demonstrates the mechanism of polyphyllin VI induced HepaRG cell apoptosis for the first time. It is expected that our findings will be helpful to provide guidelines regarding the safety of polyphyllin VI for the development of new pharmaceuticals and clinical administration.

## 4. Materials and Methods

### 4.1. Cell Culture

The human hepatoma HepaRG cell line was purchased from Guangzhou Jenniobio Biotechnology Co., Ltd., China. The cells were cultured in RPMI 1640 medium, supplemented with 10% fetal bovine serum and 100 U penicillin-streptomycin antibiotics at 37 °C in a humidified atmosphere of 5% CO_2_ and 95% air. Polyphyllin VI was dissolved in DMSO to a stock concentration of 16 mM and the final working concentration of DMSO in cell culture experiments was less than 0.1% for cell assays. Cells were treated with different dose of polyphyllin VI with or without inhibitors of cell signaling pathways.

### 4.2. Chemical Reagents and Antibodies

Polyphyllin VI (batch no. 111592-201604, purity > 97.0%) was purchased from China Food and Drug Testing Institute, Beijing, China. RPMI 1640 medium, penicillin, streptomycin, and FBS were provided by Gibco-Life Technologies, Waltham, MA, USA. MTT was product of Biotopped Life Sciences Co. Ltd., Beijing, China. LDH assay kit, DAPI assay kit, NAC, Annexin V-FITC apoptosis assay kit, ROS assay kit, MMP assay kit (JC-1), cell cycle assay kit(PI) were purchased from Beyotime (Nanjing, China). Antibodies for Bax (1:1000; rabbit polyclonal; cat. no. 5023; CST), Bcl-2 (1:1000; mouse polyclonal; cat.no. 15071; CST), p53 (1:1000; mouse polyclonal; cat. no. 2524, CST), p21 (1:1000; rabbit polyclonal; cat. no. 2947; CST), cyclin A2 (1:1000; mouse polyclonal; cat. no. 4656; CST), CDK2 (1:1000; rabbit polyclonal; cat. no. 2546; CST), cleaved caspase-3 (1:1000; rabbit polyclonal; cat. no. Ab2302; Abcam), cleaved caspase-9 (1:1000; rabbit polyclonal; cat. no. Ab2013; Abcam), cleaved caspase-8 (1:1000; rabbit polyclonal; cat. no. Ab25901; Abcam), cytochrome c (1:1000; rabbit polyclonal; cat. no. 4280; CST) and PARP (1:1,000; rabbit polyclonal; cat. no. 9532; CST), β-Actin (1:1000; rabbit polyclonal; cat. no. 4280; CST) and PARP (1:1000; rabbit polyclonal; cat. no. AC001-M; Santa).

### 4.3. Cytotoxicity Assay

MTT working solution (0.5 mg/mL) was used to investigate the cell viability. The cells were seeded at a density of 8 × 10^3^ cells per well in 96-well plates overnight. Then cells were treated with corresponding concentrations of polyphyllin VI (2, 4, 6, 8, 10, 12, 16 μM) and incubated for 24 or 48 h at 37 °C 100 μL MTT reagent was added to each well and incubated for 4 h in the dark. 100 μL DMSO was added to every well after aspirating the culture medium [43]. The absorbance of the formazan solution was measured by a Tecan Sunrise (Thermo Fisher Scientific, Waltham, MA, USA) at 490 nm. The cell viability was calculated by the ratio of the absorbance of treated cells and the control cells. HepaRG cells were incubated with different concentrations of polyphyllin VI for the assay of LDH. After 24 h treatment, the supernatant was collected to determined LDH activity with a commercial kit [44].

### 4.4. DAPI Staining

As previously described [44], morphological variation was observed by DNA staining with the DAPI dye. After incubation with polyphyllin VI for 24 h, cells were collected and washed with PBS once and added 4% paraformaldehyde for 20 min. After washing with PBS, cells were stained with a DAPI solution (2.5 μg/mL) for 10 min and washed twice with PBS. The morphological changes were accessed by inverted Olympus IX71 fluorescence microscope (Olympus Corporation, Tokyo, Japan.).

### 4.5. Apoptosis Analysis

Polyphyllin VI induced death of HepaRG was quantified using the PI and annexin V double staining and then measured by flow cytometry [45]. Briefly, cells were incubated at a density of 4 × 10^5^ cells/well in 6-well microplates and treated with several concentrations (0, 2, 4, 6, 8, 12 μM) of polyphyllin VI for 24 h. Then cells were collected and washed twice with PBS. The cell pellets were transferred to a 1.5 mL culture tube and resuspended in 295 μL binding buffer, then 5 μL annexin V-FITC and 10 μL PI were added. The tube was suspended gently and incubated for 20 min in the absence of light. Subsequently, the cells were measured immediately by flow cytometry (BD FACSCanto II, Becton Dickinson, New York, NJ, USA).

### 4.6. Measurement of Intracellular ROS

ROS generation was assessed following exposure to polyphyllin VI by 2′7′-dichlorfluorescein diacetate (DCFH-DA) staining (a membrane-permeable probe) and detected by flow cytometer in HepaRG cell line. Cells were seeded into a 6 well plate at 4 × 10^5^ cells/well for 24 h and then treated with polyphyllin VI at various concentrations of 0, 2, 4, 6, 8, 12 μM. After 24 h incubation, cells were washed thrice with PBS and harvested. Furthermore, NAC, a ROS scavenger, was pre-treated to HepaRG cells to determine the effect of ROS generation in polyphyllin VI induced apoptosis [46]. Fluorescence from 10,000 cells was measured and analyzed using flow cytometer (BD FACSCanto II, Becton Dickinson, New York, NJ, USA).

### 4.7. Detections of MMP

HepaRG cells were cultured for 24 h in 6-well plates and were then treated with polyphyllin VI for 24 h. All of the changes of mitochondrial membrane potential were measured by staining with JC-1 (Beyotime, Nanjing, China). The stained cells were detected by flow cytometry (BD FACSCanto II, Becton Dickinson, New York, NJ, USA).

### 4.8. Cell Cycle Analysis

PI cell cycle kit was selected to measure cell cycle distribution by flow cytometer [47]. HepaRG cells were incubated at a density of 4 × 10^5^ cells/well in 6-well plates and incubated for 24 h, and were then exposed to polyphyllin VI for 24 h at the concentrations of 0, 2, 4, 6, 8, 12 μM, respectively. Then, cells were suspended in a 500 μL PI/RNase staining buffer solution for 30 min, and then analyzed with a flow cytometer (BD FACSCanto II, Becton Dickinson, New York, NJ, USA). At least 10,000 cells could be counted for each measurement, and each group was executed in triplicate.

### 4.9. Western Blot Assaying

HepaRG cells were handled with polyphyllin VI for 24 h, washed thrice with PBS, and lysed on ice for 30 min using RIPA buffer [150 mM NaCl, 50 mM Tris, 1% Triton X-100, 1% sodium deoxycholate and 0.1% SDS]. ProteoExtract^®^ Cytosol/Mitochondria Fractionation Kit (Millipore, Cambridge, MA, USA) was used to isolate the mitochondrial and cytosolic fractions. The lysate was centrifuged at 12,000 rpm for 15 min to remove the insoluble protein. The protein concentrations were measured by BCA protein assay kit. Proteins were electrophoresed using 15% SDS-PAGE and transferred to a PVDF membrane. After blocking with 5% skim milk and washing with TBST for 1 h, the membranes were incubated with primary antibodies (1:1000) overnight and horseradish peroxidase-conjugated (1:1000) secondary antibodies for 1 h at 4 °C, and then washed thrice in TBST buffer. The target proteins were visualized by an enhanced ECL detection system (iNtRON Biotechnology, Seongnam, Korea).

### 4.10. Statistical Analysis

All of the experiments were performed in triplicates and the results were expressed as mean ± SEM. Data were analyzed by one-way analysis of variance (ANOVA), followed by the LSD test (SPSS statistics 17.0, Kabei Information Technology Co. LTD., Shanghai, China, 2012.). A value of *p* < 0.05 was considered to be significant.

## Abbreviations

DMSO	dimethyl sulfoxide
CYPs	cytochrome P450s
TCM	Traditional Chinese Medicine
RPS	Rhizoma Paridis saponins
ROS	Reactive oxygen species
MPT	mitochondrial permeability transition
MPTP	mitochondrial permeability transition pore
ATP	Adenosine triphosphate
RPMI	Roswell Park Memorial Institute
FBS	fetal bovine serum
MTT	3-[4,5-dimethylthiazol-2-yl]-2,5 diphenyl tetrazolium bromide
LDH	lactate dehydrogenase
PI	propidium iodide
DCFH-DA	2′7′-dichlorfluorescein diacetate
BCA	bicinchoninic acid
SDS-PAGE	sodium dodecyl sulfate polyacrylaminde
PVDF	polyvinylidene fluoride
MMP	mitochondrial membrane potential
TBST	Tris-buffered saline containing Tween-20
